# Post-transcriptional regulation of MRE11 expression in muscle-invasive bladder tumours

**DOI:** 10.18632/oncotarget.1627

**Published:** 2014-01-14

**Authors:** Rebecca M. Martin, Martin Kerr, Mark T.W. Teo, Sarah J. Jevons, Marianne Koritzinsky, Bradly G. Wouters, Selina Bhattarai, Anne E. Kiltie

**Affiliations:** ^1^ Gray Institute for Radiation Oncology and Biology, Department of Oncology, University of Oxford, Oxford, UK; ^2^ Section of Epidemiology and Biostatistics, Leeds Institute of Cancer and Pathology, Leeds, UK; ^3^ Princess Margaret Cancer Centre, University Health Network, Toronto, Canada and Department of Radiation Oncology, University of Toronto, Toronto, Canada; ^4^ Department of Histopathology and Molecular Pathology, St James's University Hospital, Leeds, UK

**Keywords:** MRE11, bladder cancer, post-transcriptional regulation, microRNA, miR-153, protein stability

## Abstract

Predictive assays are needed to help optimise treatment in muscle-invasive bladder cancer, where patients can be treated by either cystectomy or radical radiotherapy. Our finding that low tumour MRE11 expression is predictive of poor response to radiotherapy but not cystectomy was recently independently validated. Here we investigated further the mechanism underlying low MRE11 expression seen in poorly-responding patients. MRE11 RNA and protein levels were measured in 88 bladder tumour patient samples, by real-time PCR and immunohistochemistry respectively, and a panel of eight bladder cancer cell lines was screened for MRE11, RAD50 and NBS1 mRNA and protein expression. There was no correlation between bladder tumour MRE11 protein and RNA scores (Spearman's rho 0.064, p=0.65), suggesting MRE11 is controlled post-transcriptionally, a pattern confirmed in eight bladder cancer cell lines. In contrast, NBS1 and RAD50 mRNA and protein levels were correlated (p=0.01 and p=0.03, respectively), suggesting primary regulation at the level of transcription. MRE11 protein levels were correlated with NBS1 and RAD50 mRNA and protein levels, implicating MRN complex formation as an important determinant of MRE11 expression, driven by RAD50 and NBS1 expression. Our findings of the post-transcriptional nature of the control of MRE11 imply that any predictive assays used in patients need to be performed at the protein level rather than the mRNA level.

## INTRODUCTION

Muscle invasive bladder cancer can be treated by surgical removal of the bladder (cystectomy) or radical radiotherapy, with similar cure rates [1]. However, it is not currently possible to choose the better option for an individual patient, based on known clinico-pathological parameters. There is therefore an urgent need to develop predictive biomarkers in this disease. In two cohorts of radiotherapy patients, we found that patients whose tumours expressed low levels of the DNA damage signalling protein, MRE11, as measured by immunohistochemistry (IHC), had a significantly worse survival rate following radiotherapy than those expressing high levels of MRE11 (43% versus 70% 3-year cause-specific survival) [2]. Low MRE11 expression was predictive of poor response to radiotherapy, rather than being a prognostic marker in bladder cancer, as expression was not correlated with outcome in our surgical cohort. More recently, Laurberg *et al* [3] confirmed our MRE11 IHC findings in a Danish surgical and German chemoradiotherapy cohort. We observed lower MRE11 expression in tumour cells than normal urothelium [2], as seen previously in breast tumours [4, 5]; low MRE11 was associated with poorer radiotherapy outcomes in breast cancer [5].

MRE11 is a component of the MRN complex with RAD50 and NBS1, and stability of the three MRN proteins is linked, with MRE11 mutations which destabilise the MRN complex being associated with reduced RAD50 and NBS1 protein levels [6]. Re-expression of MRE11 in such mutant cells results in increased levels of MRE11, RAD50 and NBS1 proteins [6, 7]. The MRN complex contributes to the DNA damage response, detecting DNA breaks and signalling to checkpoint kinases [8]. The crystal structure of the human MRE11 core has recently been determined [9]. Through its exonuclease activity, MRE11, along with CtIP, resects DNA ends during homologous recombination [10] and promotes microhomology-mediated end-joining over conventional non-homologous end-joining [11]. MRE11 is also required for telomeric maintenance [12]. Cells defective in MRE11 expression are unusually radiosensitive [6], and treatment with the MRE11 inhibitor Mirin results in inhibition of radiation-induced phosphorylation of ATM [13, 14]. Therefore our validated IHC result was intriguing, as it was anticipated that reduced expression of proteins involved in DNA repair such as MRE11 would increase patient survival after radiotherapy, through greater tumour radiosensitivity from reduced repair of DNA double-strand breaks.

The aims of the present study were to further our understanding of the mechanisms underlying the low tumour MRE11 protein expression seen in poorly responding bladder cancer patients, as potentially this could be exploited clinically, and to determine the level of control, relative to transcription. We wished to test the hypotheses that underlying control mechanisms determine MRE11 protein expression levels, and that expression levels of NBS1 and RAD50 might also influence MRE11 expression.

## RESULTS

### MRE11 protein expression is controlled at the post-transcriptional level and correlates with expression of RAD50 and NBS1

**Figure 1:**
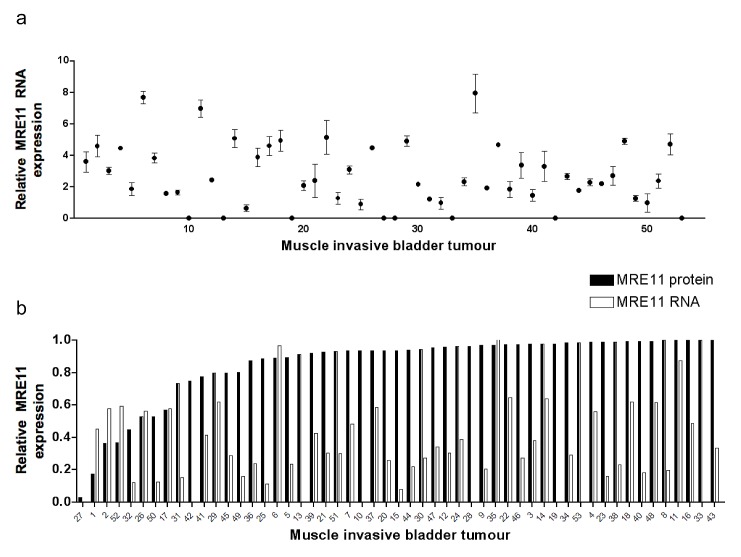
Bladder tumour MRE11 RNA and protein expression levels. a) QPCR of RNA from FFPE bladder tumours (n=53), using SDHA and ATPB as endogenous controls. Data points represent values relative to the lowest value above zero. Error bars represent the standard error of the mean (SEM) from technical duplicates. b) Tumour MRE11 QPCR and IHC scores expressed relative to the highest value for each.

To determine the range of MRE11 protein expression levels in radiotherapy patients' tumour samples and to determine whether control of expression occurs pre- or post-transcriptionally, FFPE patient tumour sections were stained by IHC for MRE11 and 0.6 mm cores taken from a homogeneous tumour area for RNA extraction. RNA of sufficient quantity and quality was extracted from 83 of 88 tumours. Fourteen tumours were excluded as the combined Ct value for both endogenous controls was greater than two standard deviations from the mean; a further 16 tumours were excluded due to inconsistencies between technical replicates. Fifty-three tumours yielded high quality data that were normalised against endogenous controls and set relative to the highest value (Figure 1). MRE11 RNA expression was below detectable levels in eight tumours, but PCR failure was ruled out as other experiments on the same plate were successful and the result was repeated on at least one other occasion. This result was therefore taken to represent extremely low MRE11 RNA expression. IHC scores were also plotted relative to the highest value and compared to QPCR results. Spearman Rank Correlation revealed no correlation between protein and RNA scores (Spearman's rho (ρ) 0.064, p=0.65), suggesting that differences in MRE11 protein expression occur as a result of post-transcriptional events.

**Figure 2:**
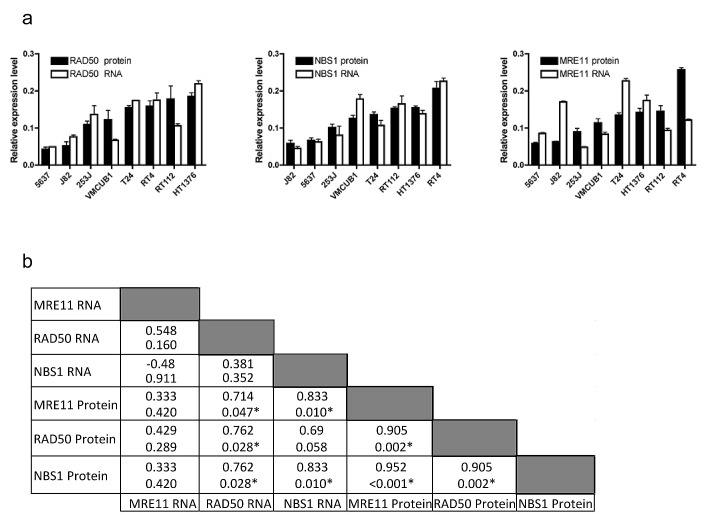
Expression of MRE11, RAD50 and NBS1 RNA and protein in eight bladder cancer cell lines a) comparison of QPCR and western blotting data for RAD50 (ρ=0.762, p=0.03), NBS1 (ρ=0.833, p=0.01) and MRE11 (Spearman's rho 0.333, p=0.42). Error bars: SEM of three experiments. b) Spearman correlation coefficients (top) with p values (bottom) for each comparison.

The same Taqman assay was used to detect MRE11 RNA expression in a panel of bladder cancer cell lines and a similar discordance was seen between RNA expression and protein expression, by western blotting (Figure 2, p=0.42, and Supplementary Figure 1). However, RNA and protein expression were correlated for both NBS1 (p=0.01) and RAD50 (p=0.03), using SYBR green. While there was no correlation between MRN complex members' mRNA expression levels, western blot quantification revealed correlations between MRE11, RAD50 and NBS1 protein expression (all p=0.002 or less), and MRE11 protein levels also correlated with both RAD50 and NBS1 mRNA levels (Figure 2b).

### microRNA-153 expression is associated with reduced MRE11 protein expression

In our clinical samples, MRE11 appeared to be controlled at the post-transcriptional level. Therefore we decided to study various factors which could be involved, namely RNA stability, protein translation initiation and protein stability. Binding of microRNAs to the 3'UTRs of mRNAs, a major post-transcriptional gene regulatory mechanism, inhibits translation of messenger RNA, by targeting it for degradation or inhibiting translation initiation.

**Figure 3:**
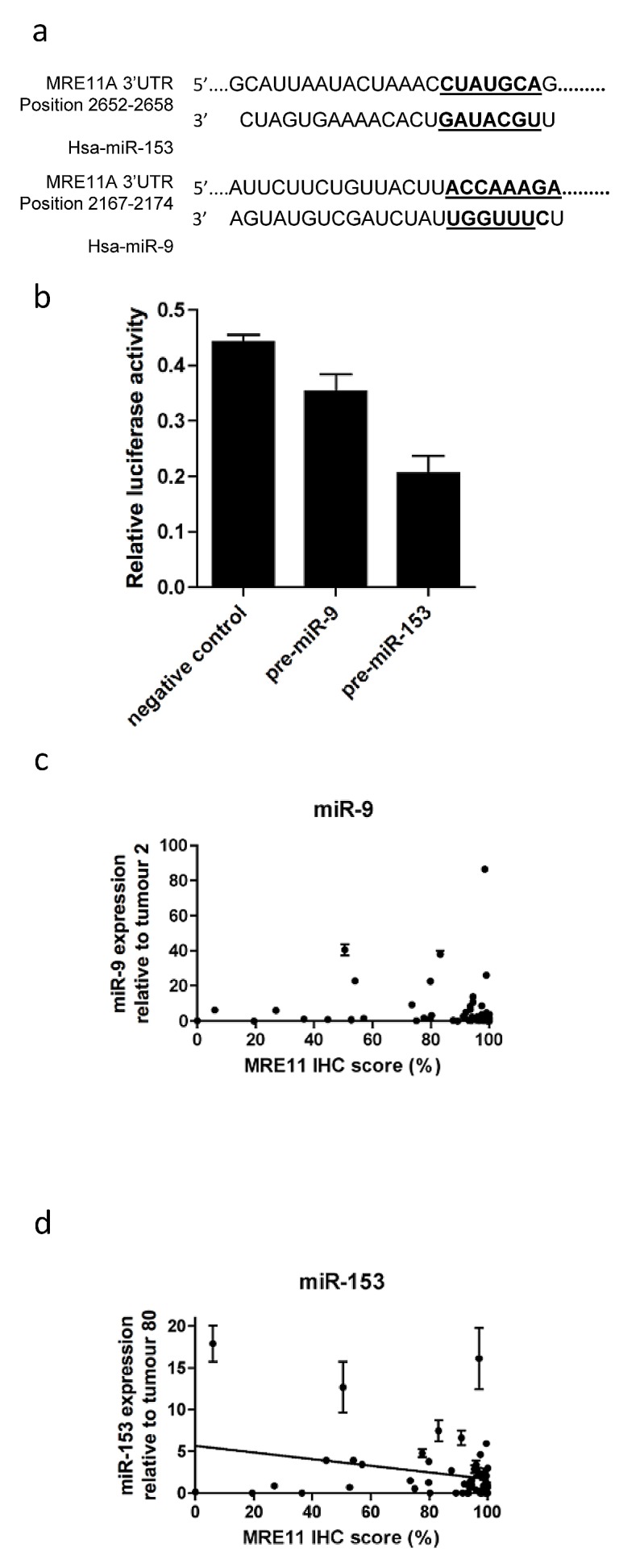
Effects of microRNA-9 and microRNA-153 on MRE11 protein expression in bladder cancer a) binding sites of miRNAs to MRE11 3'UTR; b) transfection of 5637 cells with a construct containing the MRE11 3'UTR cloned downstream of firefly luciferase, pre-miR-9, pre-miR-153 or negative control, and renilla luciferase internal control; c) correlation between microRNA-9 (n=56), and d) microRNA-153 expression (n=54) with bladder tumour MRE11 IHC protein expression. Error bars: SEM of technical duplicates.

The online database Targetscan identified miR-9 and miR-153 as having a high probability of binding to MRE11's 3'UTR (Fig 3a), and these miRNAs have higher expression in urothelial tumours than normal tissues (J Catto, personal communication, 30 Oct 2013). Therefore luciferase assays were performed in which either pre-miR-9 or pre-miR-153 was co-transfected with an MRE11 3'UTR-luciferase construct into 5637 bladder cancer cells (Figure 3b and Supplementary figure 2), chosen for transfection efficiency of both plasmid DNA constructs and small RNAs. miR-9 had a small, borderline significant effect on luciferase activity (p=0.06), whereas transfection of miR-153 markedly reduced luciferase activity (p=0.002). This functional assay suggested that miR-153 can bind the MRE11 3'UTR and inhibit gene expression, whereas miR-9 has only a relatively weak effect.

Expression of miR-9 and miR-153 was determined by QPCR in tumours using total RNA extracted from FFPE tissues from additional tumour cores, mentioned previously. miRNAs, being much smaller than mRNA can be reliably measured in FFPE material [15]. Sixty-two tumours yielded RNA of sufficient quality and quantity for analysis. Six tumours were excluded as combined levels of endogenous controls RNU44 and RNU48 were over two standard deviations from the mean, leaving 56 tumours with high quality data available. Two tumours were excluded from the miR-153 study due to inconsistencies in technical replicates or QPCR failure. In general, expression of both microRNAs was low, with higher expression in relatively few tumours (Figure 3 c-d). There was no correlation between miR-9 expression and MRE11 IHC score (p=0.14), but miR-153 expression was inversely correlated with IHC expression (ρ-0.403, p=0.003), consistent with our *in vitro* data.

### Translation initiation does not affect MRE11 expression

Although we observed a relationship between miR-153 and protein levels for MRE11, no correlation was observed with mRNA expression (data not shown). We hypothesized that miR-153 could influence protein levels by inhibiting translation initiation of MRE11 in bladder tumours. We therefore assessed whether or not protein expression of MRE11 was influenced at the level of translation initiation using the polysome assay. The T24 cell line has a lower MRE11 protein:RNA ratio (1:1.7) than the 253J cell line (1:0.5) so we hypothesised that translation initiation in T24 cells would be lower than in 253J cells. QPCR was carried out on RNA extracted from fractions separated on a sucrose gradient corresponding to those mRNAs attached to 0-1, 2-4, 5-7 or 7+ ribosomes.

**Figure 4:**
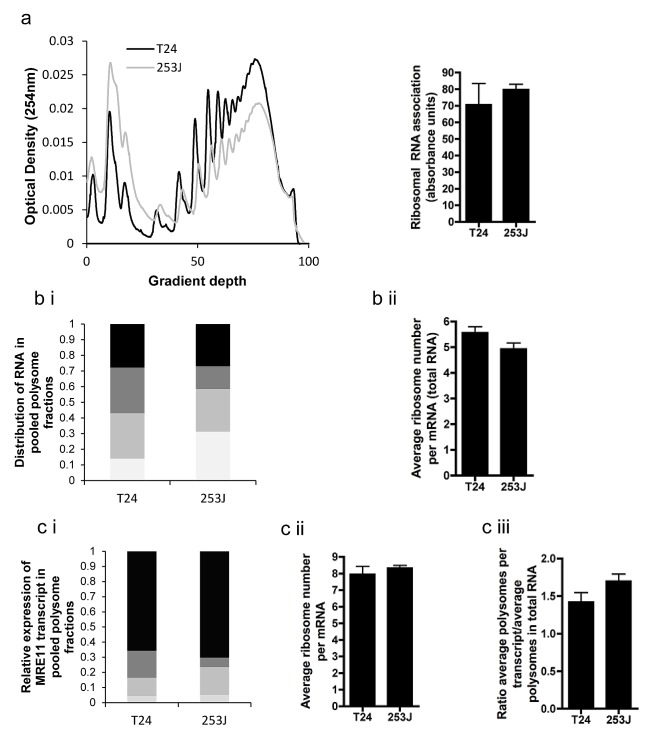
Polysome assay performed on T24 and 253J cells a) example polysome plot of overall translation, with sum of total RNA for each cell line derived from polysome plot; b)(i) Example of distribution of RNA in fraction pools corresponding to 0-1 (light grey), 2-4 (medium grey), 5-7 (dark grey) and >7 (black) ribosomes. (ii) average ribosome number per mRNA of total RNA, c(i) Example of MRE11 transcript distribution across fraction pools as determined by QPCR (legend as for b(i)), (ii) mean ribosome number per MRE11 mRNA, (iii) rate of translation of MRE11 adjusted for global differences in translation.

The overall levels of protein synthesis (percentage of ribosomes involved in translation) was similar in both lines (Figure 4a). The distribution of mRNA within the polysome gradient was also similar (Figure 4bi). The average ribosome number per mRNA transcript, determined from these distributions, was slightly lower for 253J cells although this did not reach statistical significance (Figure 4bii). In contrast to our hypothesis, QPCR demonstrated no significant differences in MRE11 translation between the two cell lines, even once adjusted to compensate for differences in global translation by normalisation against average ribosome number per mRNA transcript (Figure 4c, p=0.15). These data suggest that the discrepancy between MRE11 mRNA and protein levels cannot be explained in these two cell lines by differences in translation initiation, nor was there a difference in miR-153 levels (Supplementary Figure 4).

### Bladder cancer cell lines have generally stable MRE11, RAD50 and NBS1 mRNA and protein levels

**Figure 5:**
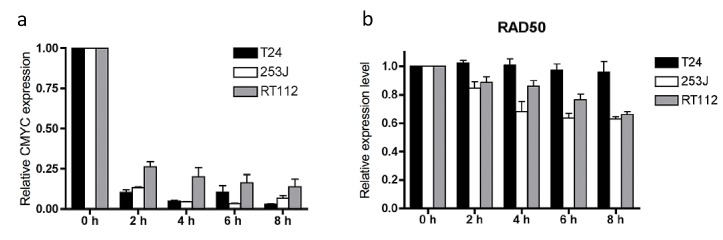
a) c-MYC RNA stability and b) RAD50 RNA stability in T24, 253J and RT112 cells Relative data expressed relative to time zero = 1.0 for each transcript. Error bars: SEM of three independent experiments.

RNA stability was measured by treatment with 1 ug/ml Actinomycin D, an inhibitor of transcription, followed by QPCR using SYBR Green. The efficacy of Actinomycin D is reflected in the rapid decrease in c-MYC RNA in all cell lines (Figure 5a). MRE11 and NBS1 were relatively stably expressed in all three lines tested (Supplementary Figure 3); RAD50 was also stable in T24 cells but RAD50 mRNA levels fell by 37.2% (p=0.0052) and 34.1% (p=0.019) in 253J and RT112 cells, respectively, by 6 hours, with half lives calculated as 229 hours and 2.45 hours. All half-lives were statistically significantly greater than those of c-Myc in individual cell lines (Figure 5b and Supplementary Table 3), and represent a general high level of stability for all three MRN transcripts.

**Figure 6:**
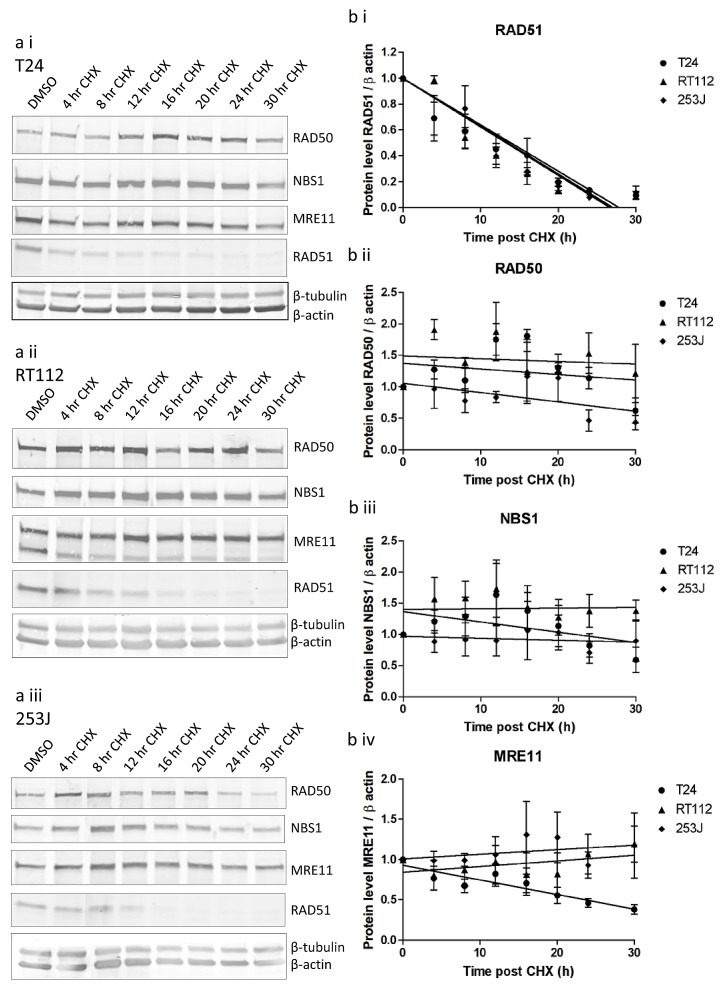
MRE11, RAD50 and NBS1 protein stability in T24, RT112 and 253J cells measured using cycloheximide assay a) western blots in (i) T24, (ii) RT112, (iii) 253J cells, b) quantification curves for (i) RAD51, (ii) RAD50, (iii) NBS1, (iv) MRE11. Relative data is expressed relative to 1.0 at time zero for each transcript. Error bars: SEM of three independent experiments.

Protein stability was determined by treating cells with 100 μg/ml cycloheximide for up to 30 hours (Figure 6). Cell death prevented study over a longer time period. RAD51 levels fell relatively rapidly, confirming cycloheximide's ability to inhibit further translation. All three proteins were significantly more stable than RAD51, with half-lives of at least 25 hours. All half-lives were statistically significantly greater than those of RAD51 in individual cell lines (Supplementary Table 3). There were no statistically significant differences in degradation of MRE11, RAD50 or NBS1 between cell lines.

## DISCUSSION

The aim of this study was to further investigate our observation that patients with low tumour MRE11 expression do worse following radical radiotherapy than high MRE11 expressors [2]. At the start of this study it was not known whether control of MRE11 expression was at the pre-transcriptional (i.e. mutation of the *MRE11* gene or epigenetic silencing of *MRE11*), transcriptional or post-transcriptional level. Post-transcriptional processes involved in control of gene expression include mRNA degradation, translation and protein degradation [16]. Also, as the three proteins act in a complex, MRE11 protein expression may be influenced by NBS1 or RAD50 expression.

To compare mRNA and protein expression, we selected a small tumour area for IHC scoring and removal of tissue cores for mRNA extraction and QPCR RNA quantification. We found that MRE11 protein and RNA expression were discordant, implying that MRE11 expression is subject to post-transcriptional control. Post-transcriptional gene regulation is widespread in the cell and accounts for up to 60% of protein abundance [17, 18]. The selection of a small tumour area was necessary, as we previously found that tumour chips from TURBT specimens frequently show heterogeneous MRE11 staining. We found no association between MRE11 protein expression in our single TURBT chip per patient and survival on Kaplan-Meier analysis (data not shown), unlike our previous study, but this reflects our inability to measure tumour heterogeneity in our single chip [19]. This highlights the need to assess all tumour areas immunohistochemically for predictive purposes.

Our clinical findings of discordant MRE11 mRNA/protein expression were backed up by the cell line data, which additionally showed concordant RAD50 protein/mRNA and NBS1 protein/mRNA levels, suggesting MRE11 expression is regulated by a post-transcriptional mechanism but not RAD50 or NBS1. Since this study began, Garner and Eastman [20] studied MRN expression in the NCI60 cell line panel, which does not include bladder cancer lines, and found strong correlations for protein expression but not mRNA expression. However, MRE11 RNA and protein expression were more highly correlated than for NBS1 and RAD50, suggesting control of MRE11 at the transcription level. In contrast, our findings suggest that in bladder cancer transcriptional control dominates for RAD50 and NBS1, but MRE11 expression is regulated at the post-transcriptional level. Garner and Eastman concluded that it was the MRN complex formation that determined protein stability. Despite the different underlying mechanisms, our data would concur with this finding. In our study *RAD50* and *NBS1* mRNA levels correlated with expression of all three proteins (borderline for *NBS1* mRNA and RAD50 protein, p=0.058), implying that these two genes are driving the formation of the protein complex, with the MRE11 protein expression then determined by its binding to the complex, with degradation of protein molecules that are not required for complex formation.

MicroRNAs are small, 22-27 nucleotide length, non-coding RNAs sequences which bind to complementary binding sites in the 3'UTR of mRNAs, resulting in mRNA degradation if there is extensive base-pairing complementarity, or inhibition of mRNA translation if complementarity is limited. Either results in post-transcriptional gene silencing [21]. Targetscan identified miR-9 and miR-153 as potential binding partners for the MRE11 3'UTR. In addition, miR-9 and mir-153 have been detected in 94% and 54% of urothelial carcinomas, respectively (J Catto, personal communication, 30 Oct 2013) with 2.7 and 2.8-fold increases in expression relative to normal urothelial tissue, and we found miR-9 expression in 100% and miR-153 in 76% of tumours. However, expression was generally low, which may limit their functional impact to a subset of tumours.

Our luciferase assays demonstrated that pre-miR-153 transfection resulted in reduced expression of the MRE11 3'UTR, which was compatible with our clinical data, where low miR-153 expression was associated with high MRE11 protein expression in clinical samples. It is biologically plausible that miR-153 binding to the MRE11 3'UTR could inhibit MRE11 protein expression, through mRNA degradation or the inhibition of translation initiation.

In mouse fibroblasts, Schwanhausser *et al* found translation rates to be the most important factor determining protein abundance (approx 55%), followed by transcription (approx 34%), with mRNA and protein stability less important [16]. Our polysome assay results suggest that the rate of translation of MRE11 may not be an important determinant of MRE11 protein expression in bladder cancer, although we only studied two cell lines. Our data support findings that mRNA and protein stability are the least important factors determining protein abundance. The actinomycin D assay revealed relatively stable mRNA levels for all three genes across three cell lines, with half lives of at least 2.45 hours compared to a mean half live of 33 minutes for RAD51, and data from our cycloheximide experiments showed that all three proteins were also relatively stable with a minimum half life of 23.5 hours compared to a mean of 12.1 hours for c-Myc (Supplementary Table 3).

Our findings are biologically plausible in terms of the MRN complex, as all three proteins are located in the nucleus and involved in the DNA damage response. For this, the fully-formed proteins are required to move rapidly to sites of damage to form foci, involved in damage recognition and repair, and there is not sufficient time for transcription and/or translation to form new protein molecules [22], so proteins with long half-lives are needed. Interestingly, RT112 cells had a lower MRE11 band, not present in the other two cell lines, which declined more rapidly than the full-length protein, and this needs to be investigated further.

Whilst we have studied a number of post-transcriptional mechanisms of MRE11 expression, we have not ruled out the possibility that transcriptional or pre-transcriptional mechanisms might be involved rarely in individual tumours. Both epigenetic and genetic changes, such as amplification of chromosome 6p22.3, are known to contribute to the development of bladder cancer [23]. Promoter hypermethylation of *MRE11* has not been detected in previous bladder cancer studies, nor have gene mutations such as the A poly(T) *MRE11* mutation at the intron 4 splice site, seen in upper urinary tract tumours with microsatellite instability [24]. This -1 to -2 frameshift mutation in a run of 11 thymidine residues results in deletion of exons 5 to 7 of *MRE11*, and reduced expression of a protein lacking exonuclease activity [25-27]. All our cell lines were screened and none carried this mutation. In the presence of such a mutation, MRE11 expression could drive the expression of the MRN complex, as the least abundant protein. Alternative *MRE11* transcripts that do not result in a fully functional protein, but which act in a dominant-negative manner, could be detected by QPCR. However, all our MRE11 primers targeted the two common transcripts 1 and 2, and our antibody, binding amino acids 182 to 582, detects both protein isoforms.

In conclusion, we have found that, in muscle-invasive bladder tumour samples and a panel of bladder cancer cell lines, MRE11 expression is regulated at the post-transcriptional level, while RAD50 and NBS1 undergo transcriptional control. We have investigated a number of post-transcriptional mechanisms, and found that miRNA-153 expression was inversely correlated with MRE11 expression, although miR-153 was only highly expressed in a few tumours, limiting its clinical applicability. MRE11-NBS1-RAD50 exists in a 2:2:2 complex [8] and it appears that RAD50 and NBS1 transcription determines the amount of MRN complex formed, with MRE11 protein levels adapting in line with complex formation.

Regarding clinically-useful biomarkers, immunohistochemistry seems the most useful test and our results highlight the importance of studying MRE11 at the protein rather than mRNA level.

## MATERIALS AND METHODS

Ethical approval was obtained from Leeds (East) Local Research Ethics Committee (studies 02/060 and 04/Q1206/62).

### Tumour processing for IHC and RNA extraction

Eighty-eight tumour blocks from pre-radiotherapy transurethral resection of bladder tumour (TURBT) samples collected from 2002 to 2009 were identified as being suitable for this project. Patients were treated with radical radiotherapy for transitional cell carcinoma of the bladder at the Leeds Cancer Centre, West Yorkshire, UK, as per Choudhury *et al* [2]. An area of each formalin-fixed paraffin-embedded (FFPE) block was identified based on the matched haematoxylin-and-eosin (H+E) slide, where more than 70% of cells were tumour cells and the area appeared homogenous, to ensure that any core taken was representative of what was seen on the H+E slide. Four cores of tissue were taken from the identified area using a 0.6 mm tissue microarray corer (Beecher Instruments Inc). Total RNA was isolated using the HP RNA paraffin kit (Roche Diagnostics Ltd) according to manufacturer's instructions, except that cores were lysed for 3 days, quantified by Nanodrop and 200 ng used in a 20 μl Taqman reverse transcription reaction (Applied Biosystems). Whole mount sections were stained for MRE11 and the area scored for percentage positive cells and intensity score as per Choudhury *et al* [2].

### Cell culture

The bladder cancer cell lines RT112 and 253J were a generous gift from Professor MA Knowles, Leeds Institute of Molecular Medicine. They had been authenticated using by extensive genomic analysis (microsatellite typing, conventional karyotypic analysis, M-FISH and array based copy number analysis). The cell lines 5637, HT1376 and VMCUB-1 were purchased from the German Collection of Microorganisms and Cell Cultures (DSMZ), and J82, T24 and RT4 were purchased from the American Type Culture Collection. RT112, 253J, 5627 and T24 cells were culture in RPMI medium with 10% fetal calf serum (FCS, Invitrogen). HT1376 and VMCUB-1 cells in DMEM and 10% FCS, J82 cells in MEM, 1% non-essential amino acids and 10% FCS and RT4 cells in McCoy's 5A medium and 10% FCS. After resuscitation, cell lines were cultured for no more than three months.

### Western blot

Cells were lysed with buffer containing 50 mM Hepes,100 mM NaCl,10 mM EDTA,1% w/v Triton, 4 mM sodium pyrophosphate, 2 mM Sodium orthovanadate, 10 mM sodium fluoride, 50 mM β-glycerophosphate pH to 7.5 with NaOH and 1% SDS then centrifuged at 14,000G for 10 minutes to obtain the total cell lysate. The BCA assay (Fisher) was used to determine protein concentration and 50 μg of protein was loaded on an 8% or 4-20% SDS-PAGE gel, transferred to a nitrocellulose and probed with antibodies against MRE11 (Abcam, AB214), NBS1 (Novus Biologicals), RAD50 (Cell Signalling, #3427), RAD51 (Santa Cruz), β-tubulin (Sigma) and β-actin (Abcam). Fluorescent secondary anti-mouse 800 and anti-rabbit 680 antibodies (Licor Biosciences) were used at 1:5,000 and visualised and quantified on an Odyssey machine.

### Real-time PCR

For quantification of MRE11 mRNA in tumour cores and the bladder cancer cell line panel, 2 μl of reverse transcription product was used in a 20 μl QPCR using Hs00271551_m1 Taqman assay with Taqman gene expression master mix. ATP5B, Hs00969569_m1, and SDHA, Hs00188166_m1, were used as endogenous controls (Applied Biosystems) [28, 29]. RNA extracted from FFPE tissue is not of the same quality as RNA extracted from cell lines but valuable QPCR data can be obtained within the limitations involved and by monitoring endogenous control signal [30].

For expression analysis of mRNA in cell lines in subsequent experiments, SYBR green PCR master mix was used with primers for MRE11:F-TGAGGAGGTAC GTCGTTTCA,TCCATCTGGCATAAATGATGA, R-GTGGAAGTTTTCCTGCTCCA;RAD50:F-GAGATTTCCCTCCTGGAACC,R-ACATCACGAAATTGCAGACG;ATP5B:F-ACCATCAAAGGATTCCAGCA,R-GCTTTTGCCACAGCTTCTTC;SDHA:F-TGGGAACAAGAGGGCATCTG,R-CCACCACTGCATCAAATTCATG;C-MYC:F-CAGCTGCTTAGACGCTGGATT,R-GTAGAAATACGGCTGCACCGA.

RNA was isolated using the RNAeasy mini kit (Qiagen). Reverse transcription was performed using the High Capacity cDNA Reverse Transcription Kit (Applied Biosystems).

### Luciferase assays

A vector containing MRE11-3'UTR cloned into the SGG_3UTR vector downstream of luc2P was purchased from Switchgear Genomics. Two hundred nanograms of plasmid vector was transfected into 5637 cells seeded at 15,000 cells per well of a 96-well plate 24 hours previously, along with 20 ng Renilla luciferase containing vector and pre-miR (miR-9 pm10022, miR-153 PM10122 and pre-miR™ miRNA Precursor Molecules - negative control#1 AM17110, Applied Biosystems) to a final concentration of 250 nM using oligofectamine. Forty eight hours later firefly and renilla luciferase activity was detected on a Polstar Omega plate reader using dual-glo luciferase assay reagents (Promega).

### MicroRNA expression

MicroRNA expression was determined by QPCR using Taqman Universal master mix (Applied Biosystems) and microRNA assays 00583 for miR-9, 001191 miR-153, (Applied Biosystems). RNU44 (assay 1094) and RNU48 (assay 1006) were used as endogenous controls to normalise expression.

### Polysome fractionation and analysis

Polysome fractionation and analysis was performed as previously described in Koritzinsky *et al* [31]. Briefly, 70% confluent cells were treated with 0.1 mg/ml cycloheximide, lysed and the lysate applied to a sucrose gradient. After centrifugation, 1 ml fractions were collected that contained RNA bound to ribosomes with increasing weight and therefore increasing ribosome number. Fractions were pooled into groups containing 0 or 1, 2 to 4, 5 to 7 and greater than 7 ribosomes, and RNA was isolated and reverse transcribed. The translation efficiency of specific genes was determined by QPCR analysis to detect the relative amount of each transcript in the pooled fraction groups. Average ribosome number per transcript was calculated and normalised against the average ribosome number for total RNA in each cell line, to adjust for differences in overall translation rate in each cell line.

### Actinomycin D assay and cycloheximide treatment

Cells (1.5 x10^6^) were seeded in 10 cm plates the day before treatment with actinomycin D (Sigma) at 1 μg/ml and harvested 2, 4, 6, and 8 h later for RNA extraction as detailed above. Two hundred nanograms of RNA was used in a 20 ul reaction and 2 ul of a 1 in 5 dilution added to a 20 μl QPCR SYBR green reaction. A DMSO carrier control was harvested after 8 hours.

Cells (3 x10^6^) were seeded in 15 cm plates the day before treatment with Cycloheximide at 100 μg/ml and harvested up to 36 h later for protein extraction, as detailed above. However, at 36 hours, cycloheximide caused cell death. A DMSO carrier control was plated at 1x10^6^ cells to avoid over-confluency and harvested at 36 h.

### Statistics

Spearman rank correlations and Student's T test were carried out using SPSS 14.0 software. Unless otherwise stated, P values represent Student's T test results.

## SUPPLEMENTARY FIGURES AND TABLES


