# Supplementary material for: Post-transcriptional regulation of MRE11 expression in muscle-invasive bladder tumours
Source: Oncotarget. 2014 Jan 14;5(4):993–1003. doi: 10.18632/oncotarget.1627 (PMC4011600; doi:10.18632/oncotarget.1627)
Supplement: Supplementary file 1 [file oncotarget-05-993-s001.docx]

**Supplementary Information**

**Supplementary Figure 1**: Expression of MRE11, RAD50 and NBS1 RNA and protein in eight bladder cancer cell lines. a) Western blot of MRE11, RAD50 and NBS1 expression, with expression levels normalised to β-tubulin as a fraction of 1; b) Western blot quantification of β-tubulin from Fig 2A as a fraction of 1. Error Bars: SEM of three independent experiments.

a

MRE11

NBS1

RAD50

β-tubulin


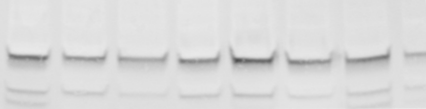

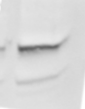

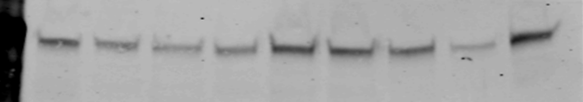

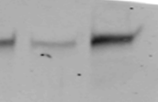

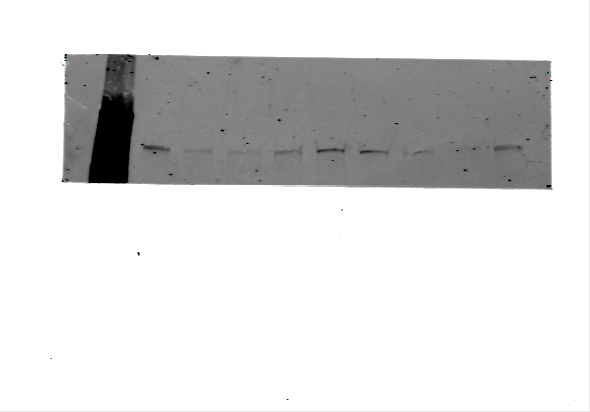

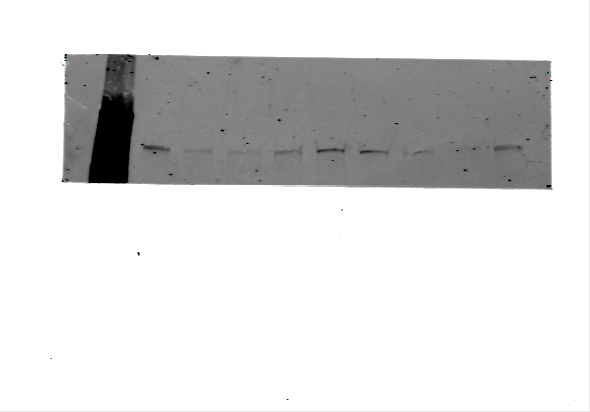

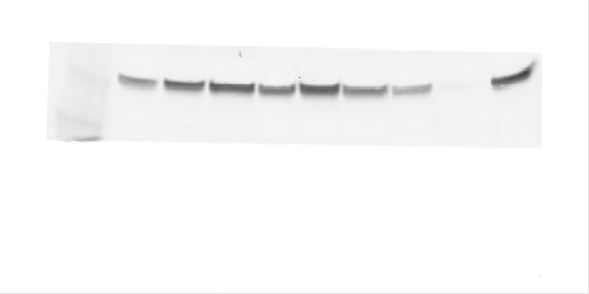

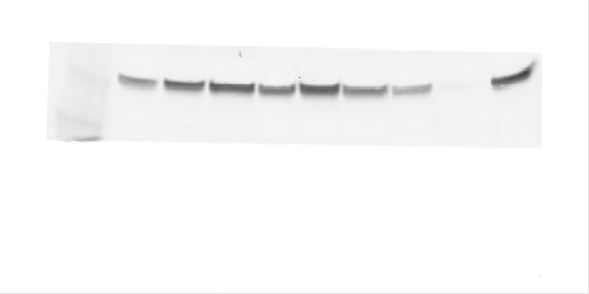


RT112 5637 J82 253J T24 HT1376 RT4 VMCUB1


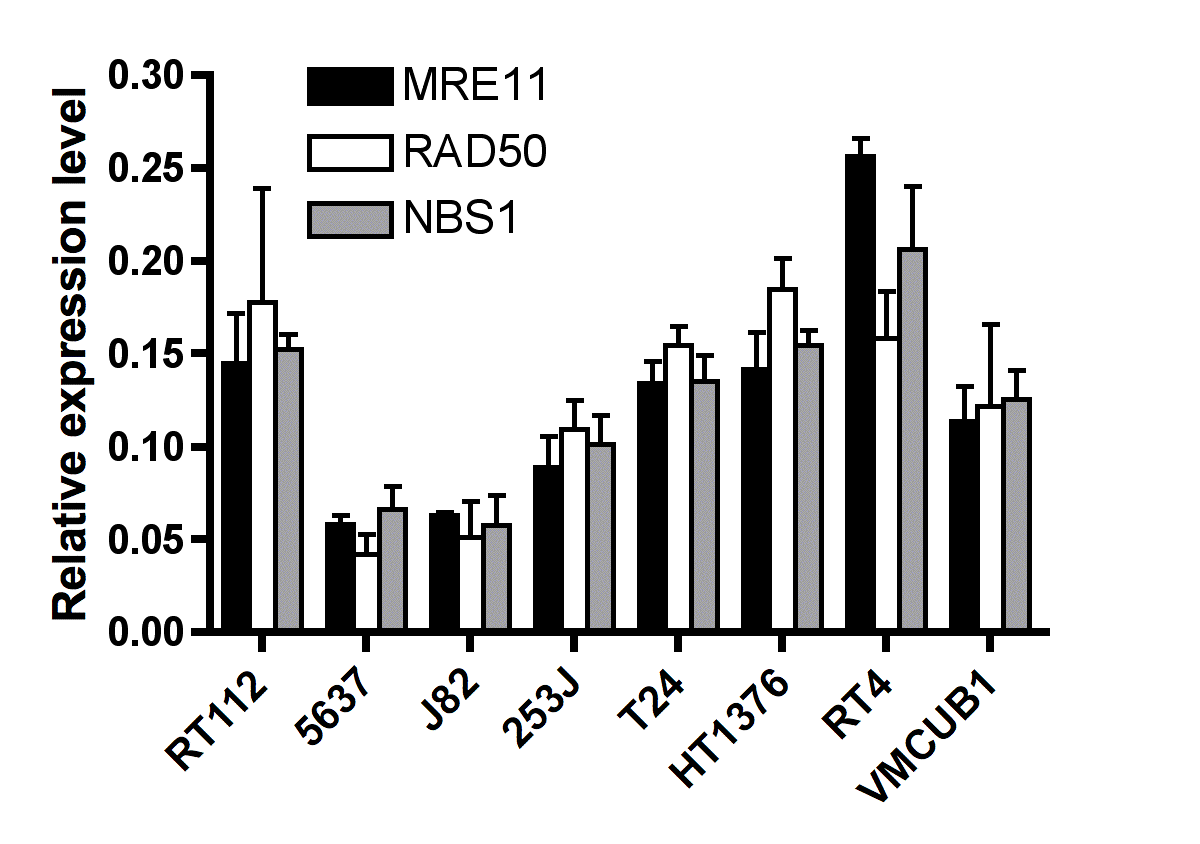


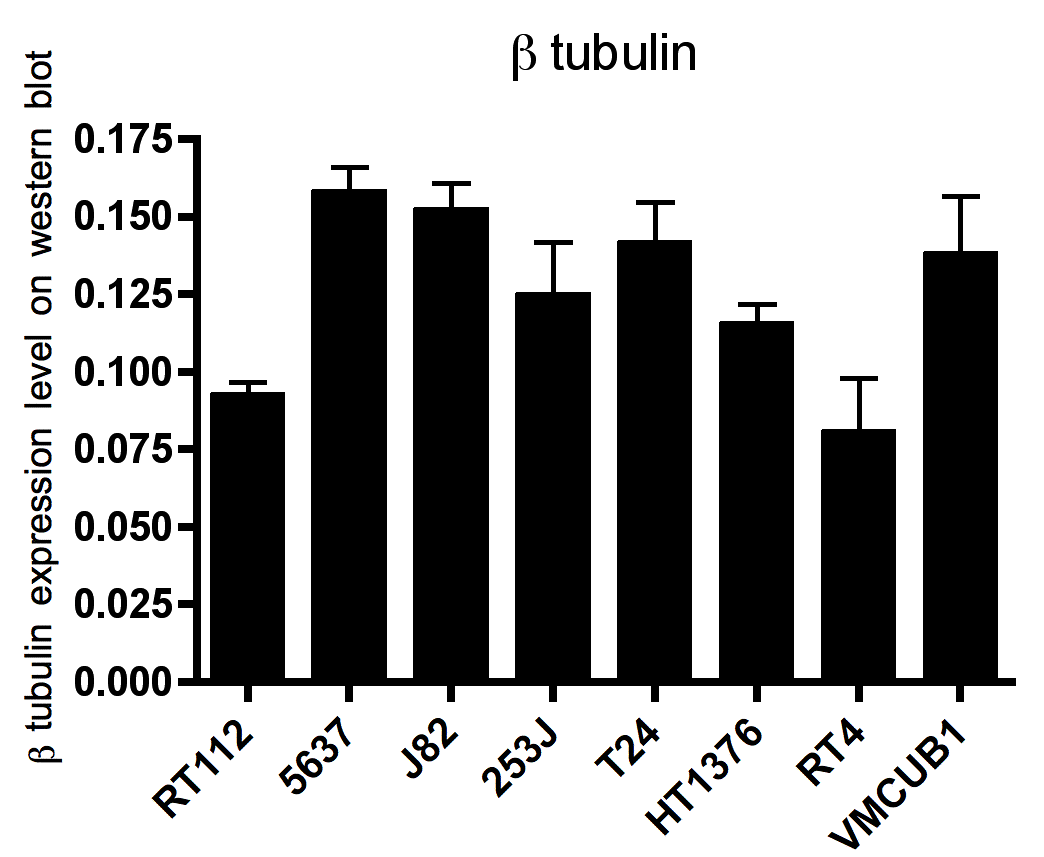
b

**Supplementary Figure 2.** Principles of the miRNA luciferase assay.

Cloning the MRE11-3’UTR downstream of luc2P gives firefly luciferase expression subject to regulation by elements that act on the MRE11 3’UTR, such as microRNAs. Full expression of luciferase occurs in transfected cells, as measured on a luminometer after treatment with a firefly luciferase substrate. On co-transfection of the vector with a microRNA, a reduced luciferase signal occurs if the microRNA in question binds to the 3’UTR.


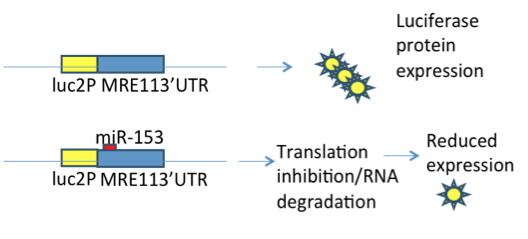


**Supplementary Figure 3.** mRNA stability for MRE11 and NBS1 in T24, 253J and RT112 cells. Relative data expressed relative to time zero = 1.0 for each transcript. Error bars: SEM of three independent experiments.


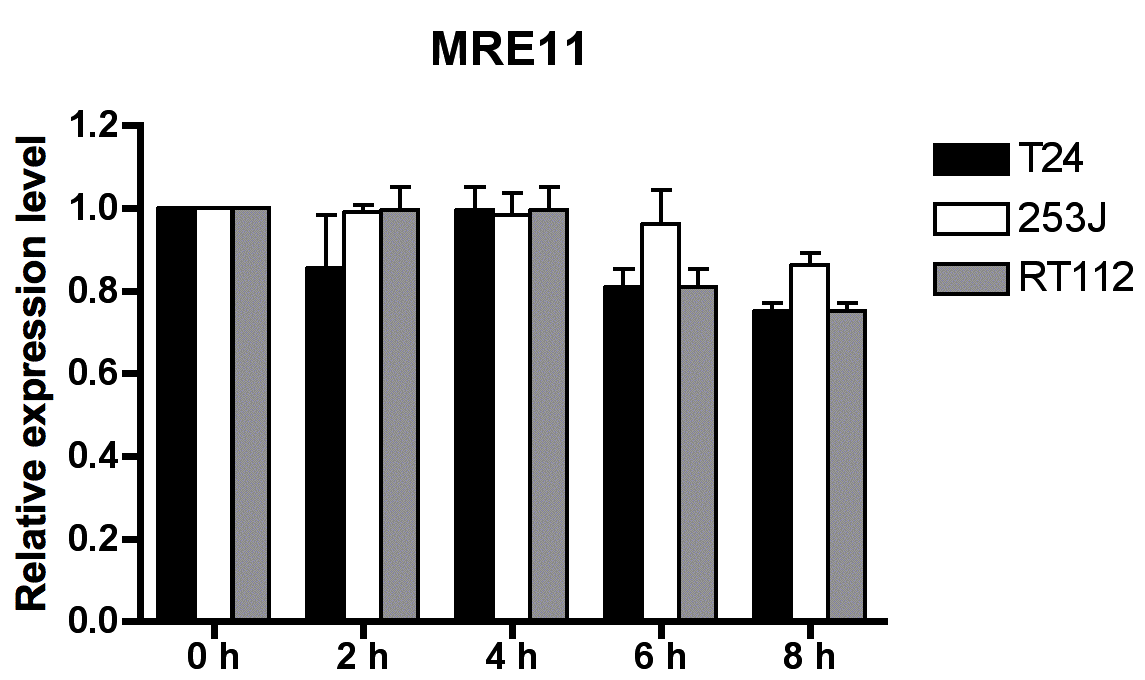


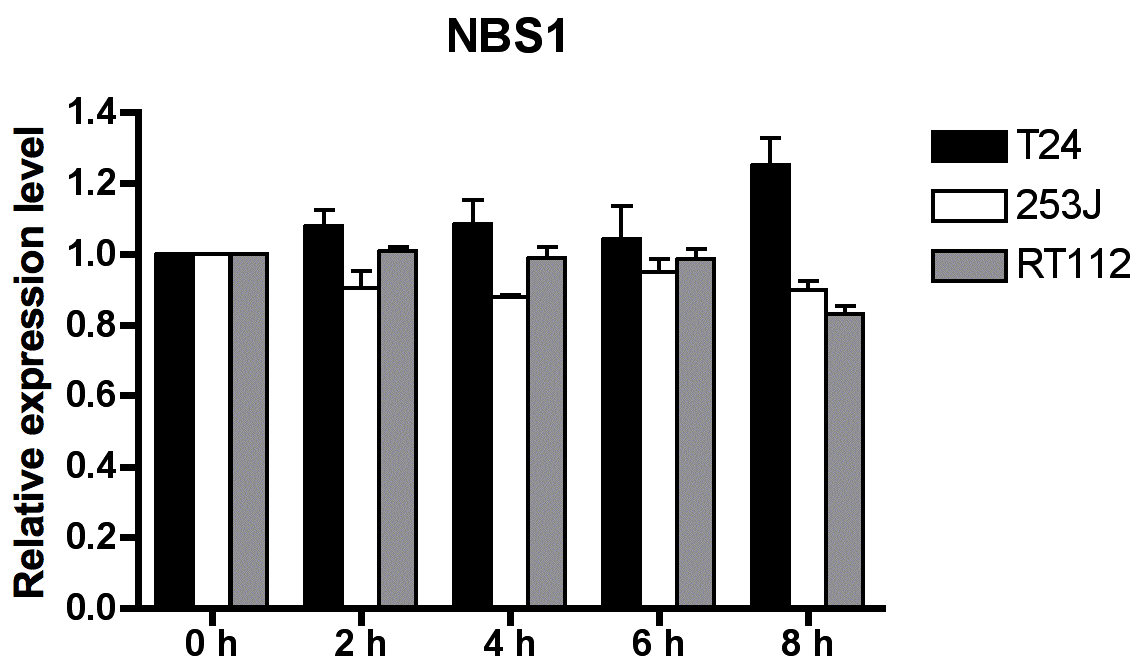


**Supplementary Figure 4.** Relative miR-153 expression in 253J and T24 cells as a fraction of 1. Error bars: SEM of three independent experiments.


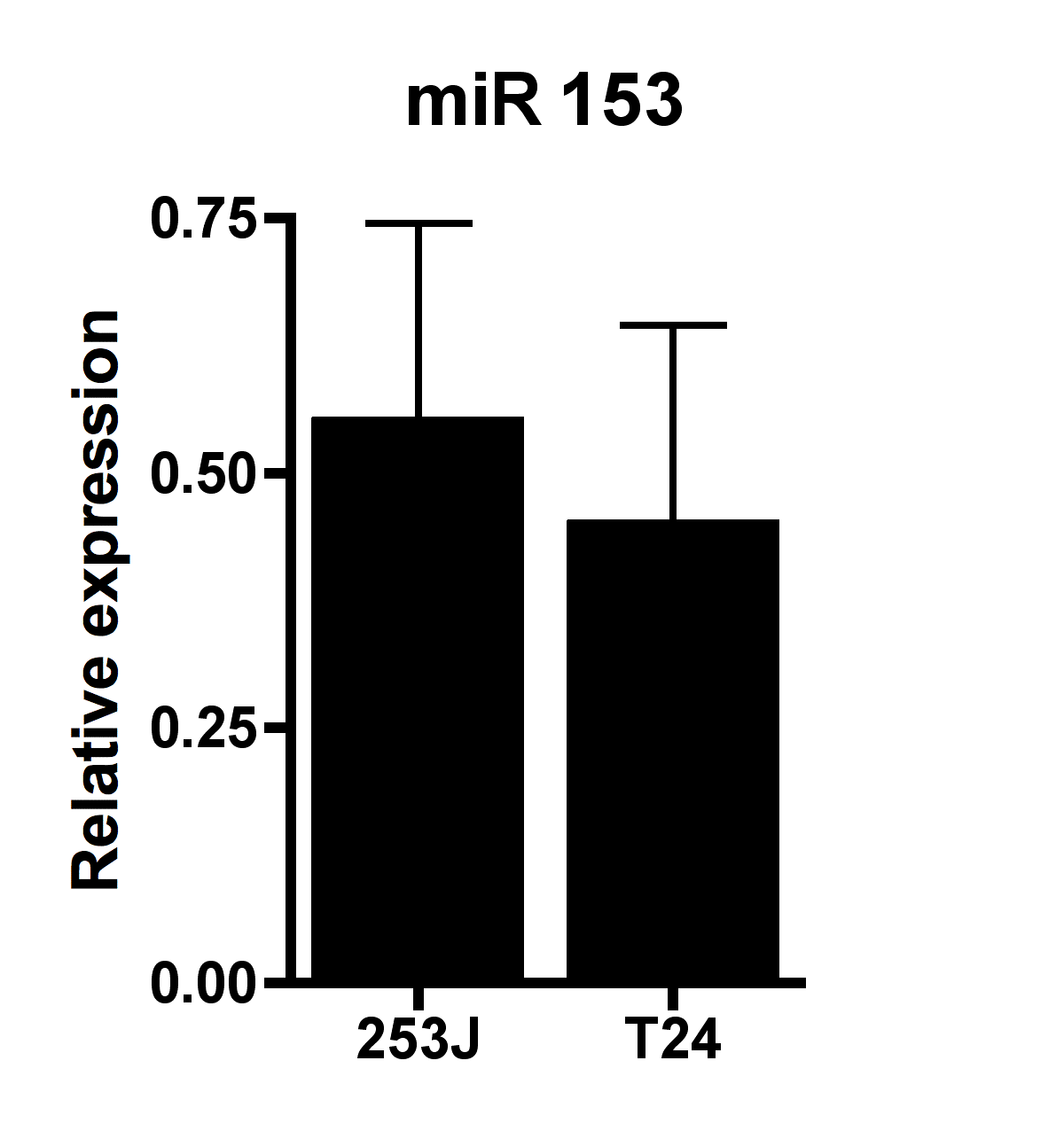


**Supplementary Table 1.** Average Ct values and standard deviations.

|  | ATP5B | | SDHA | | MRE11 | |
| --- | --- | --- | --- | --- | --- | --- |
| Tumour Number | average Ct | SD | average Ct | SD | average Ct | SD |
| 1 | 29.76 | 0.095331 | 29.06 | 0.03 | 33.74 | 0.622 |
| 2 | 32.02 | 0.116744 | 30.55 | 0.053554 | 35.26 | 0.524461 |
| 4 | 32.69772 | 0.300529 | 32.22761 | 0.315911 | 37.03965 | 0.283194 |
| 5 | 29.32971 | 0.212553 | 30.4474 | 0.01584 | 33.90519 | 0.097843 |
| 6 | 29.34271 | 0.233986 | 30.61572 | 0.131451 | 35.24741 | 0.744615 |
| 7 | 31.2372 | 0.062215 | 31.46858 | 0.050094 | 34.58297 | 0.175225 |
| 8 | 31.79595 | 0.215814 | 31.61421 | 0.018622 | 35.93925 | 0.289694 |
| 9 | 30.3283 | 0.019513 | 29.57385 | 0.535692 | 35.47913 | 0.086065 |
| 10 | 31.78 | 0.1644 | 31.53 | 0.18 | 37.11 | 0.35 |
| 12 | 31.94 | 0.06 | 31.74 | 0.162 | 0 | 0 |
| 15 | 30.29 | 0.166 | 30.99 | 0.61 | 34.01 | 0.27 |
| 16 | 30.72 | 0.094 | 30.93 | 0.082 | 35.72 | 0.053 |
| 17 | 32.91565 | 0.438506 | 33.73288 | 0.143289 | 0 | 0 |
| 18 | 32.94 | 0.000923 | 32.73 | 0.290054 | 36.66267 | 0.396399 |
| 19 | 29.7 | 0.000151 | 29.62 | 0.007626 | 36.47 | 1.08 |
| 20 | 30.87 | 0.192849 | 30.86393 | 0.091337 | 35.08368 | 0.537 |
| 21 | 31.98 | 0.10814 | 33.35574 | 0.267874 | 36.3537 | 0.468217 |
| 22 | 31.77416 | 0.079168 | 32.0446 | 0.075498 | 35.78 | 0.047478 |
| 23 | 31.93722 | 0.063501 | 31.62643 | 0.162197 | 0 | 0 |
| 26 | 31.31704 | 0.057951 | 30.97627 | 0.086028 | 36.27 | 0.517251 |
| 28 | 31.77549 | 0.162509 | 31.93725 | 0.162215 | 36.76278 | 1.434472 |
| 30 | 33.03131 | 0.58683 | 32.879 | 0.361041 | 36.76 | 0.71465 |
| 36 | 29.70715 | 0.189675 | 30.71253 | 0.749081 | 36.02498 | 0.949872 |
| 47 | 32.46124 | 0.057259 | 31.98677 | 0.259995 | 36.76606 | 0.288319 |
| 48 | 32.26889 | 0.033613 | 32.18637 | 0.026665 | 38.54629 | 1.23639 |
| 50 | 31.11018 | 0.058136 | 31.55025 | 0.211689 | 35.34038 | 0.08113 |
| 51 | 33.40986 | 0.172272 | 33.60071 | 0.211281 | 0 | 0 |
| 52 | 32.76269 | 0.06661 | 30.90312 | 0.035595 | 0 | 0 |
| 53 | 32.06022 | 0.519 | 31.72546 | 0.121044 | 35.76555 | 0.225104 |
| 54 | 29.58595 | 0.228323 | 29.79013 | 0.118814 | 34.64249 | 0.140373 |
| 55 | 27.94151 | 0.053346 | 27.66295 | 0.096864 | 33.69 | 0.189205 |
| 57 | 31.46826 | 0.215415 | 31.03066 | 0.040788 | 37.4624 | 1.289791 |
| 58 | 30.91855 | 0.158447 | 31.25829 | 0.019573 | 0 | 0 |
| 60 | 29.40602 | 0.073776 | 30.77479 | 0.080966 | 35.04098 | 0.394784 |
| 61 | 31.08395 | 0.2006 | 31.06504 | 0.061118 | 34.25674 | 0.537417 |
| 62 | 30.98334 | 0.037245 | 29.96484 | 0.210589 | 35.70639 | 0.140769 |
| 63 | 30.55761 | 0.020832 | 29.41553 | 0.031298 | 33.93922 | 0.086033 |
| 64 | 28.71468 | 0.12334 | 28.5404 | 0.009659 | 33.9102 | 0.942832 |
| 65 | 30.63419 | 0.040946 | 30.47929 | 0.032636 | 34.97368 | 0.83712 |
| 66 | 31.31134 | 0.010358 | 31.53898 | 0.000613 | 37.04211 | 0.8388 |
| 68 | 30.47641 | 0.125168 | 30.55484 | 0.041949 | 34.96128 | 0.969122 |
| 69 | 34.06009 | 0.398582 | 33.20268 | 0.276765 | 0 | 0 |
| 70 | 28.47353 | 0.291732 | 29.70055 | 0.03902 | 33.84358 | 0.252316 |
| 71 | 30.70957 | 0.303476 | 31.76563 | 0.074289 | 36.58853 | 0.082563 |
| 72 | 28.37599 | 0.073534 | 28.61209 | 0.124421 | 33.46779 | 0.307465 |
| 75 | 30.88599 | 0.013623 | 29.98445 | 0.081746 | 35.48018 | 0.202041 |
| 79 | 30.6297 | 0.101057 | 31.78174 | 0.075784 | 35.94022 | 0.735085 |
| 80 | 31.15125 | 0.24236 | 30.58752 | 0.024274 | 34.75158 | 0.140479 |
| 81 | 28.7671 | 0.119531 | 29.74767 | 0.056287 | 35.08633 | 0.479509 |
| 82 | 32.81659 | 0.391754 | 31.17725 | 0.169798 | 38.16879 | 1.827228 |
| 84 | 30.31776 | 0.26323 | 30.66507 | 0.197097 | 35.41076 | 0.665477 |
| 85 | 29.3568 | 0.674704 | 27.404 | 0.075454 | 32.31504 | 0.488026 |
| 87 | 32.34823 | 0.114616 | 31.0367 | 0.507051 | 0 | 0 |

**Supplementary Table 2.** Average Ct values and standard deviations.

| Tumour number | Average RNU44 Ct | SD | Average RNU48 Ct | SD | Average miR-9 Ct | SD | Average miR-153 Ct | SD |
| --- | --- | --- | --- | --- | --- | --- | --- | --- |
| 2 | 24.75265 | 0.187577 | 21.86752 | 0.098994 | 34.3887 | 0.72960848 | no signal | no signal |
| 3 | 24.86013 | 0.073149 | 22.24679 | 0.11323 | 33.22323 | 0.03204581 | 34.0838642 | 0.463712 |
| 4 | 23.73318 | 0.066316 | 22.23298 | 0.114126 | 34.31693 | 0.06539033 | 35.1786098 | 0.495666 |
| 5 | 25.05506 | 0.219452 | 21.18813 | 0.12192 | 31.97315 | 0.34672493 | 35.272398 | 1.416493 |
| 7 | 24.02254 | 0.040216 | 20.99198 | 0.161669 | no signal |  | no signal |  |
| 8 | 24.10597 | 0.021786 | 20.181 | 0.041369 | 30.15303 | 0.20413641 | 37.2321835 | 0.554129 |
| 9 | 22.60865 | 0.027681 | 19.51821 | 0.120101 | 36.34595 | 0.02816354 | 35.7675463 | 0.333801 |
| 11 | 25.73079 | 0.096596 | 22.51917 | 0.191724 | 30.68647 | 0.06168274 | 35.412035 | 0.01022 |
| 12 | 24.37282 | 0.228197 | 23.21419 | 0.097269 | 32.12004 | 0.18048675 | no signal |  |
| 13 | 24.5299 | 0.141272 | 21.13784 | 0.076956 | 35.38938 | 0.27591418 | no signal |  |
| 14 | 23.68611 | 0.048207 | 20.70858 | 0.097775 | 36.79279 | 0.294052 | no signal |  |
| 15 | 22.48296 | 0.154743 | 20.69191 | 0.109332 | 35.70641 | 1.25272842 | 35.2379723 | 0.305588 |
| 16 | 22.73795 | 0.245381 | 20.36638 | 0.166488 | 33.1114 | 0.28978731 | 36.1802044 | 0.689296 |
| 17 | 25.39492 | 0.191463 | 23.23115 | 0.081962 | 34.07285 | 0.44223306 | no signal |  |
| 18 | 25.31142 | 0.123042 | 22.82113 | 0.082677 | 36.76821 | 3.08961351 | 36.1127396 | 0.438484 |
| 19 | 23.98099 | 0.113474 | 22.10952 | 0.177249 | 35.9235 | 0.91160194 | 36.8724976 | 0.026305 |
| 20 | 22.84837 | 0.279589 | 21.88824 | 0.097548 | 31.45939 | 0.2935106 | no signal |  |
| 21 | 25.20307 | 0.080388 | 22.67945 | 0.03158 | 34.59098 | 0.12050087 | 35.4141254 | 0.164431 |
| 22 | 22.6783 | 0.119672 | 21.97432 | 0.112459 | 31.80491 | 0.23155443 | no signal |  |
| 23 | 22.76997 | 0.113721 | 21.98253 | 0.092889 | 32.14506 | 0.22757628 | no signal |  |
| 27 | 23.22575 | 0.064096 | 21.24099 | 0.053954 | 26.87507 | 0.05162549 | 36.3609467 | 0.387816 |
| 37 | 25.66915 | 0.150691 | 24.28296 | 0.054828 | 33.39269 | 0.56238866 | 34.0748539 | 0.421174 |
| 38 | 26.63978 | 0.074415 | 24.78546 | 0.118203 | 36.22922 | 0.39840033 | no signal |  |
| 39 | 24.92952 | 0.137388 | 23.03733 | 0.143247 | 31.97231 | 0.21251857 | no signal |  |
| 40 | 25.42795 | 0.277616 | 22.39852 | 0.08747 | 30.28197 | 0.11491765 | 38.3140888 | 0.412964 |
| 41 | 26.34351 | 0.156286 | 24.30134 | 0.084621 | 31.05823 | 0.22366491 | 34.9171963 | 0.818463 |
| 42 | 23.89639 | 0.082233 | 21.77523 | 0.096857 | 35.94624 | 0.98404597 | 35.7807255 | 0.365549 |
| 49 | 23.55809 | 0.189989 | 21.78333 | 0.060512 | 28.50297 | 0.14689761 | 33.0281487 | 0.522463 |
| 52 | 23.17951 | 0.065622 | 21.36245 | 0.164252 | 35.48713 | 0.37049575 | 34.1694908 | 0.617898 |
| 53 | 24.32264 | 0.213096 | 21.4347 | 0.167825 | 33.41702 | 0.24571141 | 35.8019409 | 0.766574 |
| 54 | 23.54823 | 0.201481 | 21.55954 | 0.170371 | 30.28759 | 0.07336363 | 35.2043858 | 0.325827 |
| 55 | 22.60129 | 0.112735 | 21.25483 | 0.225078 | 29.78014 | 0.0062701 | 34.5933533 | 0.27039 |
| 57 | 24.71457 | 0.006085 | 21.48339 | 0.166896 | 34.50787 | 0.24206883 | no signal |  |
| 58 | 23.37167 | 0.0205 | 20.17505 | 0.005876 | 34.95815 | 0.01920278 | 33.4478912 | 0.183294 |
| 59 | 25.5223 | 0.178353 | 21.54179 | 0.180821 | 36.11678 | 0.17320804 | no signal |  |
| 60 | 23.60575 | 0.021754 | 21.08147 | 0.191782 | 34.34959 | 0.04761987 | 35.8235232 | 2.355043 |
| 61 | 23.49788 | 0.051207 | 24.87082 | 0.052823 | 35.01853 | 0.957366 | 33.4342906 | 0.807538 |
| 62 | 22.18745 | 0.077712 | 24.86167 | 0.121101 | 36.02646 | 0.3576885 | 35.3519783 | 0.474915 |
| 64 | 23.76959 | 0.150642 | 24.61174 | 0.285443 | 33.83862 | 0.08271032 | 36.1362762 | 0.245755 |
| 65 | 26.61091 | 0.166992 | 22.73391 | 0.124649 | 33.46761 | 0.12612585 | 34.5617104 | 1.754594 |
| 66 | 21.83163 | 0.153405 | 21.18571 | 0.063969 | 36.58569 | 0.025717 | 34.6278954 | 0.832346 |
| 68 | 23.62729 | 0.263644 | 21.25101 | 0.028859 | 32.79107 | 0.39740122 | 34.6343409 | 1.195699 |
| 69 | 22.02948 | 0.137072 | 20.02264 | 0.060441 | 33.85886 | 0.60263924 | 32.0290674 | 0.347145 |
| 70 | 23.19046 | 0.104096 | 20.11759 | 0.073248 | 32.05662 | 0.210448 | 35.7941418 | 0.188902 |
| 71 | 22.73292 | 0.065211 | 20.71824 | 0.211007 | 31.63733 | 0.1336748 | 35.1632703 | 0.503889 |
| 72 | 22.69634 | 0.119005 | 21.95143 | 0.168811 | 28.89728 | 0.04928001 | 35.0936337 | 1.199886 |
| 73 | 24.20692 | 0.191696 | 20.24594 | 0.11273 | 30.7411 | 0.13942268 | 33.5669899 | 0.126519 |
| 74 | 23.75925 | 0.153472 | 21.23003 | 0.072983 | 32.19421 | 0.31962821 | 35.9390736 | 0.482632 |
| 75 | 23.85296 | 0.103387 | 20.79038 | 0.090269 | 31.44814 | 0.12562085 | 33.8134422 | 0.52152 |
| 77 | 22.23421 | 0.172308 | 19.91835 | 0.066743 | 33.98454 | 0.49714669 | 32.134304 | 0.169014 |
| 78 | 26.72014 | 0.192494 | 25.32316 | 0.066152 | 33.31099 | 0.05627312 | Fail |  |
| 79 | 22.07613 | 0.153609 | 21.04123 | 0.119455 | 33.2978 | 0.16130172 | 37.5823154 | 1.378589 |
| 80 | 22.44548 | 0.16026 | 20.06995 | 0.107761 | 33.1439 | 0.25920441 | 34.5207043 | 0.178446 |
| 81 | 23.36503 | 0.196128 | 21.19525 | 0.005159 | 31.70399 | 0.1059971 | 34.0162468 | 0.553015 |
| 82 | 26.28273 | 0.171833 | 23.2784 | 0.04704 | 36.36328 | 0.28282221 | 35.4696178 | 0.072617 |
| 88 | 20.57297 | 0.215357 | 20.23073 | 0.050988 | 33.77109 | 0.17315861 | no signal |  |

**Supplementary Table 3.** a) Relative mRNA levels were quantified by qPCR from samples treated with 1 μg/ml Actinomycin D. Half lives were calculated from curves fitted to a one phase exponential decay model. Half lives are hours, figures in brackets are P values for comparison with RAD51; b) Relative protein levels were quantified by western blotting from samples treated with 100 μg/ml cycloheximide. Half lives were calculated from curves fitted to a one phase exponential decay model. Half lives are hours, figures in brackets are P values for comparison with c-Myc.

a)

|  | **T24** | **RT112** | **253J** |
| --- | --- | --- | --- |
| **MRE11** | 79 (0.0183) | 79 (0.0181) | 391 (0.01167) |
| **RAD50** | 229 (0.0163) | 229 (0.0171) | 2.45 (0.0172) |
| **NBS1** | 216 (0.0166) | 161 (0.017) | n/a (0.0171) |
| **RAD51** | 0.44 | 0.68 | 0.57 |

b)

|  | **T24** | **RT112** | **253J** |
| --- | --- | --- | --- |
| **MRE11** | 23.5 (0.0033) | n/a (0.0143) | n/a (0.0035) |
| **RAD50** | 98 (0.002) | 225 (0.0005) | 74 (0.0143) |
| **NBS1** | 53 (0.0011) | n/a (0.0005) | n/a (0.0042) |
| **RAD51** | 12.8 | 10.6 | 12.8 |
